# *RSD1* Is Essential for Stomatal Patterning and Files in Rice

**DOI:** 10.3389/fpls.2020.600021

**Published:** 2020-11-30

**Authors:** Qi Yu, Liang Chen, Wenqi Zhou, Yanhuang An, Tengxiao Luo, Zhongliang Wu, Yuqi Wang, Yunfeng Xi, Longfeng Yan, Suiwen Hou

**Affiliations:** Key Laboratory of Cell Activities and Stress Adaptations, Ministry of Education, School of Life Sciences, Lanzhou University, Lanzhou, China

**Keywords:** stomatal development, stomatal density, *RSD1*, *OsSDD1*, dehydration avoidance, rice

## Abstract

Stomatal density is an important factor that determines the efficiency of plant gas exchange and water transpiration. Through forward genetics, we screened a mutant *rice stomata developmental defect 1* (*rsd1-1*) with decreased stomatal density and clustered stomata in rice (*Oryza sativa*). After the first asymmetric division, some of the larger sister cells undergo an extra asymmetric division to produce a small cell neighboring guard mother cell. Some of these small cells develop into stomata, which leads to stomatal clustering, and the rest arrested or developed into pavement cell. After map-based cloning, we found the protein encoded by this gene containing DUF630 and DUF632 domains. Evolutionary analysis showed that the *DUF630/632* gene family differentiated earlier in land plants. It was found that the deletion of *RSD1* would lead to the disorder of gene expression regarding stomatal development, especially the expression of *stomatal density and distribution 1* (*OsSDD1*). Through the construction of *OsSDD1* deletion mutants by CRISPR-Cas9, we found that, similar to *rsd1* mutants, the *ossdd1* mutants have clustered stomata and extra small cells adjacent to the stomata. *OsSDD1* and *RSD1* are both required for inhibiting ectopic asymmetric cell divisions (ACDs) and clustered stomata. By dehydration stress assay, the decreased stomatal density of *rsd1* mutants enhanced their dehydration avoidance. This study characterized the functions of *RSD1* and *OsSDD1* in rice stomatal development. Our findings will be helpful in developing drought-resistant crops through controlling the stomatal density.

## Introduction

Stomata are small valves in the epidermis of plants for gas exchange between plants and the environment and play essential roles in regulating photosynthesis and water use efficiency (Hetherington and Woodward, 2003). Proper stomatal density and patterning are very important for the growth of plants. There are great differences in stomatal patterning and development processes between monocotyledons and dicotyledons. In *Arabidopsis*, stomata are constantly generated in different positions of the epidermis during leaf development. The asymmetric entry division of some protodermal cells named meristemoid mother cell (MMC) initiates the stomatal lineage and produces a larger daughter cell called stomatal lineage ground cell (SLGC) and a smaller meristemoid (M). The M can undergo asymmetrically amplifying divisions to renew itself and generate more SLGCs. Then, the M converts into the guard mother cell (GMC). The GMC divides equally to form a pair of guard cells (GCs). The SLGCs can differentiate into pavement cells or divide asymmetrically to produce a new M oriented away from preexisting stomata or stomatal precursors (Bergmann and Sack, 2007).

Most of crops belong to grasses, and they have a great impact on food security (Godfray et al., 2010; Elert, 2014). In contrast to the scattered pattern of *Arabidopsis* leaves, stomata in graminoid grasses (monocots) are distributed in files. Stomatal development in rice consists of six stages. Epidermal cells that acquired lineage fate undergo an asymmetric entry division to produce two daughter cells, a small cell, and a large sister cell (Stages I and II) (Stebbins and Shah, 1960; Luo et al., 2012; Raissig et al., 2016; Wu et al., 2019; McKown and Bergmann, 2020). Since the absence of a stem-cell-like meristemoid stage in the rice stomatal development, the small cell is named GMC (Stebbins and Shah, 1960; Nunes et al., 2020). The GMC induces the polarization of the subsidiary mother cell (SMC), which then divides asymmetrically to produce a subsidiary cell (SC) and a pavement cell (Stage III and Stage IV) (Cartwright et al., 2009; Facette et al., 2015). After that, GMCs divide symmetrically to produce a pair of GCs (Stage V). Finally, the four-cell stomatal complex is formed (Stage VI) (Stebbins and Shah, 1960).

The stomatal lineage cell fate transformation mechanism has been well studied in *Arabidopsis*. Three basic helix-loop-helix (bHLH) family transcription factors SPEECHLESS (SPCH), MUTE, and FAMA control the consecutive MMC-M-GMC-GC cell fate transitions (Ohashi-Ito and Bergmann, 2006; MacAlister et al., 2007; Pillitteri et al., 2007; Lampard et al., 2008; Chen et al., 2020). These specified cell state transitions require another two paralogous bHLH transcription factors, INDUCER OF CBF EXPRESSION1 (ICE1) and SCREAM2 (SCRM2), to form heterodimers with SPCH, MUTE, and FAMA (Kanaoka et al., 2008). In addition, the cell fate transition from GMC to GC is regulated by FOUR LIPS (FLP) and MYB88, two partially redundant R2R3 MYB transcription factors (Lai et al., 2005; Lee et al., 2014).

Recently, the molecular mechanisms that promote stomatal development in grasses are gradually elucidated. In grasses, the new factors OsSCRs/OsSHRs control the initiation of stomatal lineage cells, and the formation of SCs has been reported recently (Schuler et al., 2018; Wu et al., 2019). The core factors regulating stomatal fate transformation have similar but different functions. OsSPCH1/2 control formation of stomatal files (Raissig et al., 2016; Wu et al., 2019). *OsMUTE* is expressed in early stage of GMCs and moves to SMC to regulate SC formation. In addition, OsMUTE is involved in the direction of GMC division (Raissig et al., 2017; Wang et al., 2019; Wu et al., 2019). OsFAMA influences SMC division and differentiation of mature stomata (Liu et al., 2009; Wu et al., 2019). OsICE1 and OsICE2 influence the initiation of stomatal lineage, GMC transition, SMC division, and the differentiation of mature stomata (Raissig et al., 2016; Wu et al., 2019). The *OsFLP* regulates the direction of GMC division (Wu et al., 2019). In addition, an A2-type cyclin; OsCYCA2;1 positive regulates entry division in stomatal file (Qu et al., 2018).

The stomatal patterning in *Arabidopsis* follows the one-cell-spacing rule; that is, two stomatal complexes are separated by at least one non-stomatal cell to ensure a reasonable stomatal density and a proper stomatal patterning in different environmental conditions (Lau and Bergmann, 2012; Dow et al., 2014; Qi and Torii, 2018). Epidermal patterning factors (EPFs) include negative regulators *EPF1/2* and *EPFL4-6*, and a positive regulator *EPFL9/STOMAGEN* regulates stomatal density (Hara et al., 2007, 2009; Hunt and Gray, 2009; Abrash and Bergmann, 2010; Sugano et al., 2010; Niwa et al., 2013). These ligands bind to the receptor complex consisting of ERECTA family receptor kinase [RLK; ER, ERECTA-LIKE1 (ERL1), and ERL2] and TOO MANY MOUTHS (TMM) (Shpak et al., 2005; Lee et al., 2012, 2015). Downstream of the receptors is a mitogen-activated protein kinase (MAPK) cascade, which is composed of YODA and MKK4/5/7/9 and MPK3/6 to inhibit SPCH activity (Bergmann et al., 2004; Wang et al., 2007; Lampard et al., 2008). The predicted serine protease STOMATAL DENSITY AND DISTRIBUTION1 (SDD1) also negatively regulate stomatal patterning and density by genetically acting upstream of TMM (Berger and Altmann, 2000; Von Groll et al., 2002). The function of SDD1 in dicotyledonous plants is conserved. Overexpression of tomato *SchSDD1-like* in cultivated tomato plants decreased the stomatal index and density (Morales-Navarro et al., 2018).

The grass stomatal patterning stands in line and also abides by the one-cell-spacing rule that two stomatal complexes are separated by at least one pavement cell. The role of EPFs is conserved in stomatal development. In rice and wheat, the overexpression of *OsEPF1/2* and *TaEPF1/2* has been shown to increase water use efficiency by reducing stomatal density (Hughes et al., 2017; Caine et al., 2019; Dunn et al., 2019). *OsEPFL9* can promote stomatal development, and knocking down *OsEPFL9* reduces stomata density in rice (Lu et al., 2019). In addition, *BdYODA1* in *Brachypodium distachyon* involved in maintaining stomatal lineage fate asymmetry and loss of *BdYODA1* results in large sister cells obtain stomatal fate (Abrash et al., 2018). In maize, the overexpression of *ZmSDD1* results in stomatal density decrease of 30% and enhances the drought resistance (Liu et al., 2015).

In this study, we identified a novel stomatal mutant from EMS mutants’ library. This mutant exhibits clustered stomata and reduced stomatal file density and was named as *rice stomata developmental defect 1* (*rsd1*). Detailed analysis of stomatal development process indicated that larger sister cell of entry division underwent excessive asymmetric division in *rsd1-1*. Map-based cloning showed that *RSD1* encoded a protein also named REL2, which functions in controlling leaf rolling. The quantitative reverse transcription–quantitative polymerase chain reaction (RT-qPCR) result indicated that the expression of *OsSDD1* was significantly down-regulated in *rsd1* mutants. Knockout of *OsSDD1* produced similar stomatal phenotype with *rsd1* mutants, clustered stomata, and extra small cells adjacent to the stomata. *OsSDD1* and *RSD1* are both required for inhibiting ectopic ACDs and clustered stomata. More importantly, the looss of *RSD1* decreased stomatal density and resulted in higher dehydration avoidance.

## Materials and Methods

### Plant Materials and Growth Conditions

Rice (*Oryza sativa* L. *japonica* cv. Zhonghua 11, ZH11) was used as the wild type in this study. The *rsd1-1* mutant with clustered stomata was screened from the M2 generation of EMS mutant library and then back-crossed into ZH11 three times prior to use. The *rel2* mutant was acquired from Kun-ming Chen’s lab (Supplementary Table 1). The seedlings were grown initially on Murashige and Skoog (MS) medium under 16:8 h, light–dark cycles for 5–7 days and then cultivated in the glasshouse at Lanzhou University (Gansu, China), with a 12 h photoperiod, 60–80% relative humidity, and a day/night temperature of 32°C/22°C.

### Dental Resin Impression and Stomatal Density

The dental resin impression method was used to screen mutants *rsd1-1*, *rsd1-2*, *rel2*, *sdd1-1*, and *sdd1-2* with clustered stomata. Fully expanded fourth and fifth rice leaves were used to impress the abaxial side, and the detailed impression procedures and stomatal density statistics were performed through methods in our previous report (Luo et al., 2012).

### Imaging and Microscopy Analysis

For confocal imaging, the FM4-64 was captured using a Nikon (A1R+Ti2-E) confocal microscope. The base of the fifth leaf was cut into 0.5 cm pieces stained in FM4-64 solution. The strain method was performed according to our previous report (Wu et al., 2019). Images of the leaf were used for statistical analyses.

### Map-Based Cloning of *RSD1*

Plants with the clustered stomata were isolated as recombinants from F2 plants of a cross between the *rsd1-1* (*O. sativa* L. *japonica* cv. Zhonghua 11, ZH11) and 9,311 (*O. sativa* L. *indica*) hybrids were selected using dental resin impressions for mapping. The published RM-series rice simple sequence repeat markers^1^ were used to map the mutant gene. The locus was roughly mapped between RM228 and RM590 on the short arm of chromosome 10 by the primary location. Subsequently, the locus was fine mapped onto the ∼440 kb region between two new development markers X-02 and X-08 using 76 homozygote mutants (Supplementary Table 1). The markers were designed by Primer Premier 5.0 and the genomic sequence acquired from the Gramene. The candidate gene *RSD1* was identified by sequence analysis of all genes on the region.

### Phylogenetic Tree Construction

The genes containing DUF630 and DUF632 domains were identified from the databases JGI, *Marchantia polymorpha* (Bowman et al., 2017), *Physcomitrella patens* (Lang et al., 2018), *Arabidopsis thaliana* (Lamesch et al., 2012), *Medicago truncatula* (Young et al., 2011), *Solanum lycopersicum* (Tomato Genome, 2012), *O. sativa* (Ouyang et al., 2007), *Zea mays* (Schnable et al., 2009), *B. distachyon* (Vogel et al., 2010), *Zostera marina* (Olsen et al., 2016), and *Brachypodium stacei* (Gordon et al., 2020) by using the reciprocal BLAST technique with RSD1 protein sequence. The program BLASTP had an *e*-value cutoff of 1-E30. These sequences were further verified using Simple Modular Architecture Research Tool (SMART) protein analyzing software (Letunic and Bork, 2017). Sequences that were confirmed by both methods were used for further analyses. Eventually, the genes from this species were used for phylogenetic analyses in this study. Full-length amino acid sequences were aligned using CLUSTALW2 (Larkin et al., 2007). The neighbor-joining (NJ) (Saitou and Nei, 1987) tree was constructed by using the Molecular Evolutionary Genetics Analysis version 5.0 (MEGA 5). The tree nodes were evaluated by bootstrap analysis with 1,000 replicates. Branches with bootstrap values less than 50% were collapsed. The evolutionary tree is displayed by Interactive Tree of Life^2^ (Letunic and Bork, 2019).

### Generation of Mutant Plants by CRISPR/Cas9

The Vector pBGK032 to construct CRISPR/Cas9 line was performed from our previous report (Wu et al., 2019). The targeting sequences of RSD1 and OsSDD1 were selected (Supplementary Table 2). The designed targeting sequences were inserted into pBGK032 vector to produce CRISPR/Cas9 plasmids as described previously (Wu et al., 2019). The vectors were transformed into rice cultivars ZH11 as described previously (Nishimura et al., 2006). The transgene lines were extracted genomic DNA and PCR amplification acquired target sequences (Supplementary Table 1). The PCR products were sequenced and analyzed by CRISPR-GE^3^ (Xie et al., 2017). The mutant lines used in our experiments were predicted resulting truncated protein.

### Real-Time PCR Analysis

The method used for extracting the total RNA and RT has been described previously. For quantitative real-time PCR, we used a TB Green Premix Ex Taq (Takara Bio, Inc.) and a StepOnePlus Real-Time PCR System (Applied Biosystems) running a standard programme (Supplementary Table 1). For each real-time PCR experiment, individual samples had three biological replicates per experiment, and all experiments were repeated at least three times.

### Dehydration Response Analysis: Water Deficit Shock Treatment

For the measurement of water loss from leaves, 8 weeks old fully expanded leaves of rice wild-type plants ZH11 and *rsd1* mutants (*rsd1-1* and *rsd1-2*) were excised and placed on weighing paper with three replicates. All samples were dried slowly under 22°C and 50% relative humidity. The weight was measured every half hour. The percentage of the sample weight at each time point relative to the initial weight was the water loss weight. Three independent experiments were performed.

## Results

### *rsd1-1* Exhibits Clustered Stomata and Reduced Stomatal Files

**FIGURE 1:**
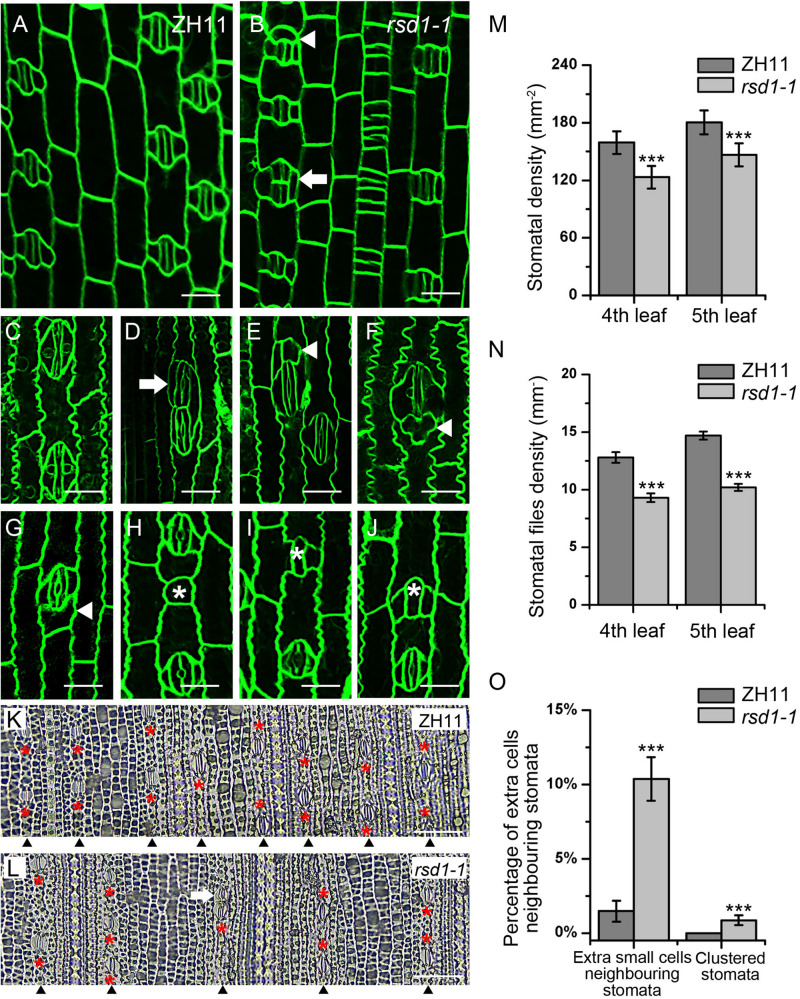
*rsd1-1* mutant showed clustered stomata and an extra small cell neighboring stoma. **(A)** Developing stomata of ZH11. **(B)** Developing stomata of *rsd1-1.* **(C)** Mature stomata of ZH11. **(D)** Two-cluster stomata sharing one subsidiary cell. **(E)** A stoma with three subsidiary cells and a small cell. **(F)** Five-cell stomatal complex. **(G)** An extra small cell neighboring stoma. **(H)** A small cell arrested in GMC stage. **(I)** Arrested GMC with one subsidiary cell. **(J)** Arrested GMC with both sides of subsidiary cell. Bars, 10 μm. The white arrowheads indicated ectopic extra small cells neighboring stomata. The white arrows indicated clustered stomata. The white asterisks indicated arrested GMCs. The red asterisks indicated stomata. **(K)** The stomatal files of ZH11. **(L)** The stomatal files of *rsd1-1*. The white arrowheads indicated stomatal files. Bars, 50 μm. **(M)** Quantification of the stomatal density at mature stage. **(N)** Quantification of the stomatal files at mature stage. **(O)** Quantification of the extra small cells neighboring stomata and clustered stomata at the fifth leave. The error bars indicated the mean ± SEM, *n* = 20; ***P < 0.001 by Student’s *t*-test.

By screening EMS mutants’ library generated from *O. sativa japonica* cultivar ZH11, we identified a stomatal mutant with clustered stomata, decreased stomatal density, and files (Figures 1A,B,K–N). We named the mutant as *rice stomata developmental defect 1* (*rsd1*). In wild type, stomata strictly comply with the principle of “one-cell-spacing rule,” that is, two stomatal complexes had to be separated by at least one pavement cell (Figures 1A,C). In *rsd1-1*, clustered stomata were observed, and some of them co-use a SC (Figures 1B,D). In addition, we observed small cells neighboring some stomata in stomatal file (Figures 1B,E–G). Some of the small cells were able to induce an extra SC, but they would not develop into mature stomata (Figure 1E). Some of small cells seemed to obtain the fate of pavement cell with lobes (Figures 1F, G). Occasionally, we observed that some GMCs exited stomatal lineage before or after inducing unilateral or bilateral SCs (Figures 1H–J), suggesting that *RSD1* is required for promoting GMC to differentiate into mature stomata. Statistical analysis revealed that the percentage of clustered stomata or extra small cell neighboring stomata significantly increased (Figure 1O). We observed that the stomatal density and stomatal file density of *rsd1-1* decreased in the same position and phyllotaxis of leaf blade (Figures 1K–N). Together, these results indicated that *RSD1* regulates stomatal distribution pattern and density.

### *RSD1* Is Essential for the Differentiation of Large Sister Cells Into Pavement Cell to Establish the Stomatal Patterning

**FIGURE 2:**
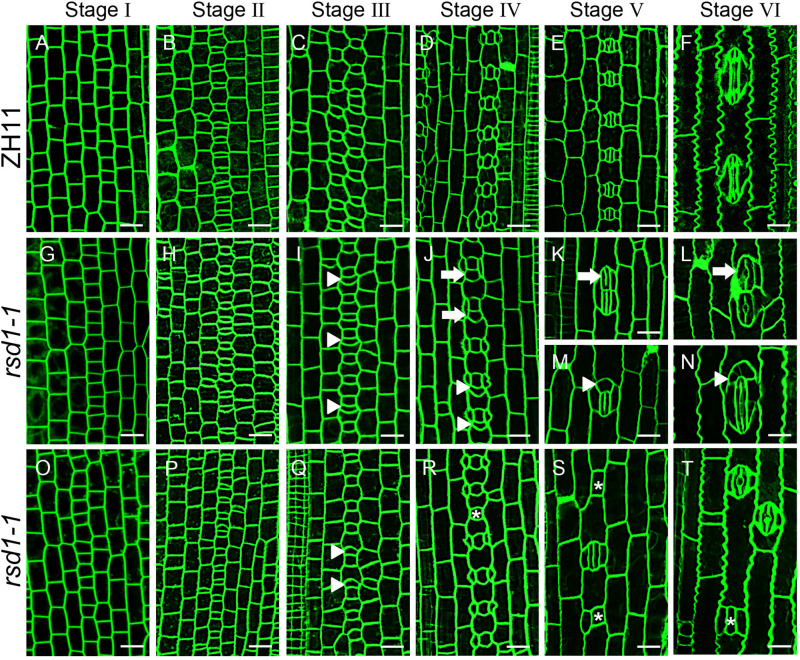
Stomatal developmental process in *rsd1-1* mutant. **(A–T)** Confocal images of six main stomatal developmental stages in *rsd1-1* mutant. There was no significant difference between *rsd1-1* and ZH11 at stage I and stage II **(A**,**B**,**G**,**H**,**O**,**P)**. Extra divisions in large sister cells resulting in extra small cells neighboring GMCs at stage III in *rsd1-1* mutant **(I**,**Q)**. Two-cluster GMCs sharing one subsidiary cell and arrested GMC at stage IV in *rsd1-1* mutant **(J**,**R)**. Clustered stomata, five-cell stomatal complex and arrested GMCs at stage V in *rsd1-1* mutant **(K**,**M**,**S)**. Morphogenesis and differentiation of clustered stomata, five-cell stomatal complex and arrested GMC at stage VI in *rsd1-1* mutant **(L**,**N**,**T)**. The white arrowheads indicated ectopic extra small cells neighboring stomata. The white arrows indicated clustered stomata. The asterisks indicated arrested GMCs. Bars, 10 μm.

The stomatal patterning and density of grasses are established in a very small area at the base of the leaf. Stomatal development in grasses consists of six stages (Figures 2A–F) (Luo et al., 2012; Raissig et al., 2016; Wu et al., 2019). At stage II, the stomatal lineage cells initiate entry division to generate a smaller GMC and a larger sister cell that will differentiate into a pavement cell (Stebbins and Shah, 1960; Raissig et al., 2016; Wu et al., 2019). The direct differentiation of large sister cells into pavement cells determines the establishment of stomatal patterning in stomatal files.

The stomatal development stages of *rsd1-1* were observed. The stomatal development of *rsd1* mutant at stages I and II were regular (Figures 2G,H,O,P). At stage III, some of the larger sister cells underwent an extra asymmetric division to produce an extra small cell neighboring GMC (Figures 2I,Q). At stage IV, a large number of extra small cells neighboring GMC were produced (Supplementary Figure S1). We observed that some SCs flanked a GMC and its neighboring extra small cell (Figure 2J). Occasionally, we also observed that the GMCs failed to induce SC formation (Figure 2R). At stage V, a few extra small cells neighboring GMC can divide equally to form paired GCs, resulting in clustered stomata (Figure 2K), and the rest of the extra small cells neighboring GMC exit stomatal linage (Figure 2M). In addition, the arrested GMCs were occasionally observed (Figure 2S). At stage VI, the abnormal stomata differentiated into mature stomata and form disrupted pattern (Figures 2L,N,T). These observations indicated that *RSD1* is required to prevent ACD in large sister cells’ reentry stomatal lineage.

### Map-Based Cloning of *RSD1* Gene

**FIGURE 3:**
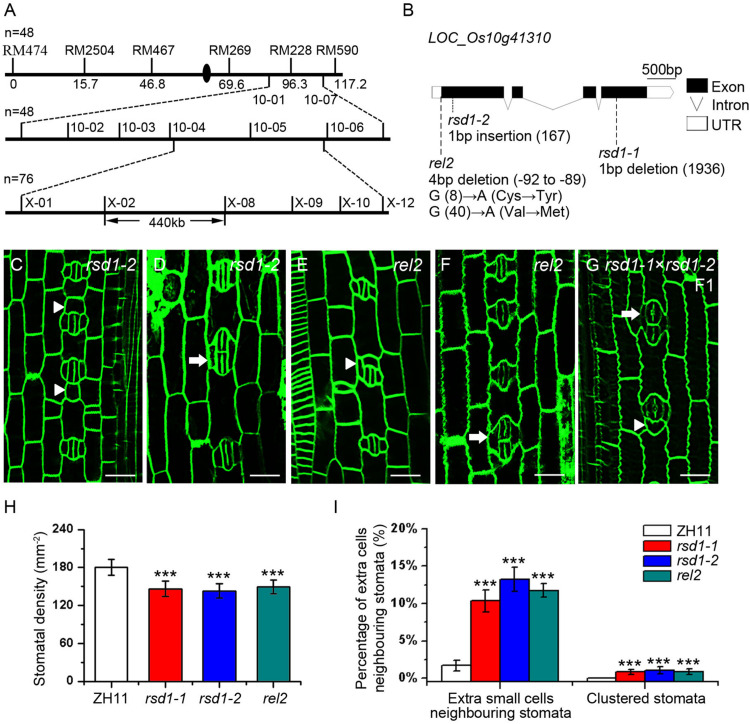
Map-based cloning of *rsd1-1* and the phenotype of *rsd1-1* allelic mutants *rsd1-2* and *rel2*. **(A)** *RSD1* was localized between X-02 and X-08 on chromosome 10. *n* represents sample size. **(B)** Gene structure and mutant sites of candidate gene *LOC_Os10g41310* (*RSD1*). Black boxes indicated the exons, lines indicated the introns, and white boxes represented untranslated region (UTR). The *rsd1-1* had a 1 bp deletion in the fourth exon. The *rsd1-2* had a 1 bp insertion in the first exon. The *rel2* had a 4 bp (GGAG) deletion in 5′-UTR and two mutations (G to A and G to A) in the first exon. **(C**–**G)** Confocal images of developing stomata in *rsd1-2*, *rel2*, and F1 generation plant of a cross between *rsd1-1* and *rsd1-2*. **(C)** An extra small cell neighboring stoma in *rsd1-2*. **(D)** Two-cluster stomata sharing two subsidiary cells in *rsd1-2*. **(E)** An extra small cell neighboring stoma in *rel2*. **(F)** Two-cluster stomata sharing two subsidiary cells in *rel2*. **(G)** An extra small cell neighboring stoma and clustered stomata in F1 generation plant of a cross between *rsd1-1* and *rsd1-2*. Bars, 10 μm. The white arrowheads indicated ectopic extra small cells neighboring stomata. The white arrows indicated clustered stomata. **(H)** Quantification of the stomatal density at mature stage. **(I)** Quantification of the clustered stomata and extra small cells neighboring stomata at the fifth leaf. The error bars indicated the mean ± SEM, *n* = 10; ***P < 0.001 by Student’s *t*-test.

Our genetic analysis showed that the clustered stomata of *rsd1-1* were caused by a single recessive mutation. To identify the mutated gene, the map-based cloning strategy was used, and the candidate gene locus was mapped in a 440 kb region between two newly developed molecular markers (X-02 and X-08) in chromosome 10 (Figure 3A). Using whole-genome sequencing, we found a 1 bp deletion at the fourth exon of *LOC_Os10g41310*, resulting in a premature transcription termination (PTT) (Figure 3B). The rice genome contains only one copy of the *RSD1* gene, which is predicted to encode a protein consisting of 767 amino acid residues. This gene is also named *Rolled and Erect Leaf 2* (*REL2*), which is involved in the control of leaf rolling in rice (Yang et al., 2016).

To confirm whether the stomatal phenotypes of *rsd1-1* were caused by the mutation of *LOC_Os10g41310*, we created an additional frameshift mutation that is 1 bp insertion in the first exon, resulting in a PTT by clustered, regularly interspaced short palindromic repeats–associated nuclease 9 (CRISPR/Cas9). We named this mutant *rsd1-2* (Supplementary Figure S2). It exhibited similar stomatal phenotypes with *rsd1-1* (Figures 3C,D,I). In addition, similar to *rsd1-1* and *rsd1-2*, the allelic mutant *rel2* produced clustered stomatal and small cell neighboring stomata (Figures 3E,F,I), and the stomatal density also decreased (Figure 3H). F1 generation plants of a cross between *rsd1-1* and *rsd1-2* show similar phenotypes with *rsd1* mutants (Figure 3G). These results indicated that the mutation of the *LOC_Os10g41310* is responsible for stomatal development defects of *rsd1* mutants.

### Evolutionary Analysis of RSD1 in Plants

**FIGURE 4:**
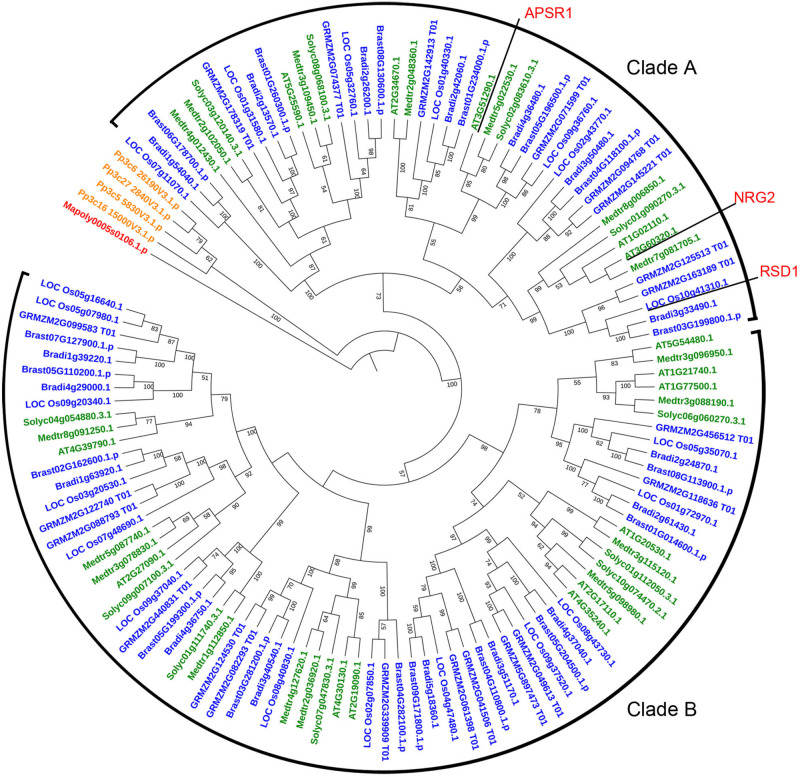
Phylogenetic relationship of proteins containing DUF630 and DUF632 domain of RSD1 in 9 land plants. The protein sequences for constructing phylogenetic tree were identified from the databases Phytozome V12 by using the reciprocal BLAST technique with rice RSD1 protein sequence. The phylogenetic tree was constructed by the NJ method in MEGA 5. Bootstrap values for 1,000 replicates are given in nodes as percentages. Amino acid sequences were used from *Marchantia polymorpha* (1 sequence), *Physcomitrella patens* (4 sequences), *Arabidopsis thaliana* (15 sequences), *Medicago truncatula* (17 sequences), *Solanum lycopersicum* (11 sequences), *O. sativa* (20 sequences), *Zea mays* (21 sequences), *Brachypodium distachyon* (17 sequences), and *Brachypodium stacei* (18 sequences).

*RSD1* encodes a protein containing DUF630 and DUF632 domains (Supplementary Figure S4). The BLASTp analysis indicated that there was no homolog of *RSD1* in green alga *Coccomyxa subellipsoidea*. In liverwort *M. polymorpha*, there was only one copy of *RSD1*. In moss *P. patens*, there were four putative paralogs. In dicots and monocots, we found lots of homologous genes contained DUF630 and DUF632 domains, such as *M. polymorpha* (1 sequence), *P. patens* (4 sequences), *A. thaliana* (15 sequences), *M. truncatula* (17 sequences), *S. lycopersicum* (11 sequences), *O. sativa* (20 sequences), *Z. mays* (21 sequences), *B. distachyon* (17 sequences), and *B. stacei* (18 sequences). In order to determine the evolutionary relationship between the *RSD1* proteins with different species, we performed multiple sequence alignment and generated an NJ phylogenetic tree for *RSD1* proteins from nine land plants (Figure 4). These genes were divided into clade A containing 45 proteins and clade B containing 74 proteins. Phylogenetic analysis indicated that the homologous genes diverged early in the evolution of land plants (Figure 4). Pairwise sequence alignment revealed that RSD1 shows 51.1% amino acid sequence identity with *Arabidopsis AtNRG2*, which plays a key role in nitrate regulation (Xu et al., 2016). Another gene *APSR1* in this family is required for root meristem maintenance (Gonzalez-Mendoza et al., 2013). These limited results indicated that this gene family had important functions in the process of plant growth and development, and the exploration of the gene function of this new gene family would be able to understand the mechanism of plant growth and development.

The number of DUF630/632 family genes in angiosperm is more than liverwort and moss remarkable (Figure 4), indicating that this gene family is growing in richness. The high number of *DUF630/632* family genes in angiosperm indicated that *DUF630/632* gene duplication might be important, which was associated with their abundance of function. In marine angiosperm *Z. marina*, the stomatal development genes (*SPCH*, *MUTE*, *FAMA*, *FLP*, *TMM*, *SDD1*, *EPF1*, *EPF2*, *EPFL9*) are absent, consisting of its phenotype without stomata (Olsen et al., 2016). However, *Z. marina* possesses two *RSD1* genes (Supplementary Figure S3). These results suggest that *RSD1* is not only related to stomatal development, but also is important for plant growth.

### *RSD1* Is Required for Proper Expression of Stomatal Development-Related Genes

The *RSD1*/*REL2* is dominantly expressed in the younger leaf blades (Supplementary Figure S5; Yang et al., 2016), suggesting that this gene is involved in stomatal development.

**FIGURE 5:**
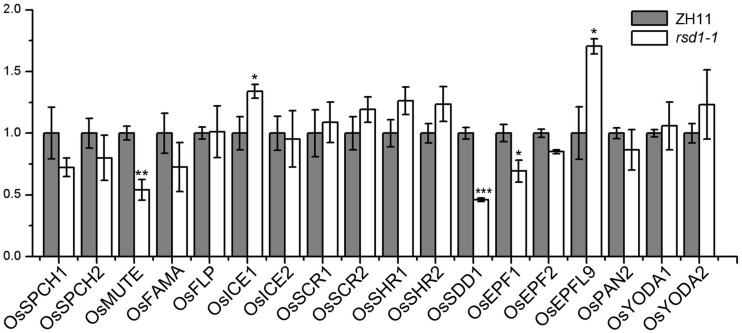
Expression analysis of stomatal development regulation genes in *rsd1-1* mutant. Analyzing the expression of stomatal development relative genes in *rsd1-1* mutant by RT-qPCR. *OsUBQ10* was used as the internal control. The error bars indicated mean ± SD, *n* = 3; ***P < 0.001. **0.001 < *P* < 0.01. *0.01 < *P* < 0.05 by Student’s *t*-test.

To investigate the relationship between *RSD1* and stomatal development, we detected the transcript abundance of 18 important stomatal development genes by RT-qPCR in the base of young leaves in *rsd1-1* (Figure 5). The expression of *OsMUTE*, *OsEPF1*, and *OsSDD1* was down-regulated in *rsd1-1* mutant, whereas the expression of *OsICE1* and *OsEPFL9* was upregulated (Figure 5). There were no significant changes in other stomatal development genes (Figure 5). The expression of *OsMUTE* and *OsSDD1* was also significantly decreased in *rsd1-2* (Supplementary Figure S6). Most small cell neighboring stomata cannot develop into stomata, and some of the GMCs arrested in the mutants. Thus, we suspected that these cells may not express *OsMUTE*, which may lead to the downregulation of *OsMUTE* in the *rsd1* mutants. These results suggest that *RSD1* is required for proper expression of stomatal development-related genes.

### *OsSDD1* Mutants Exhibit Similar Stomatal Phenotype to *rsd1*

**FIGURE 6:**
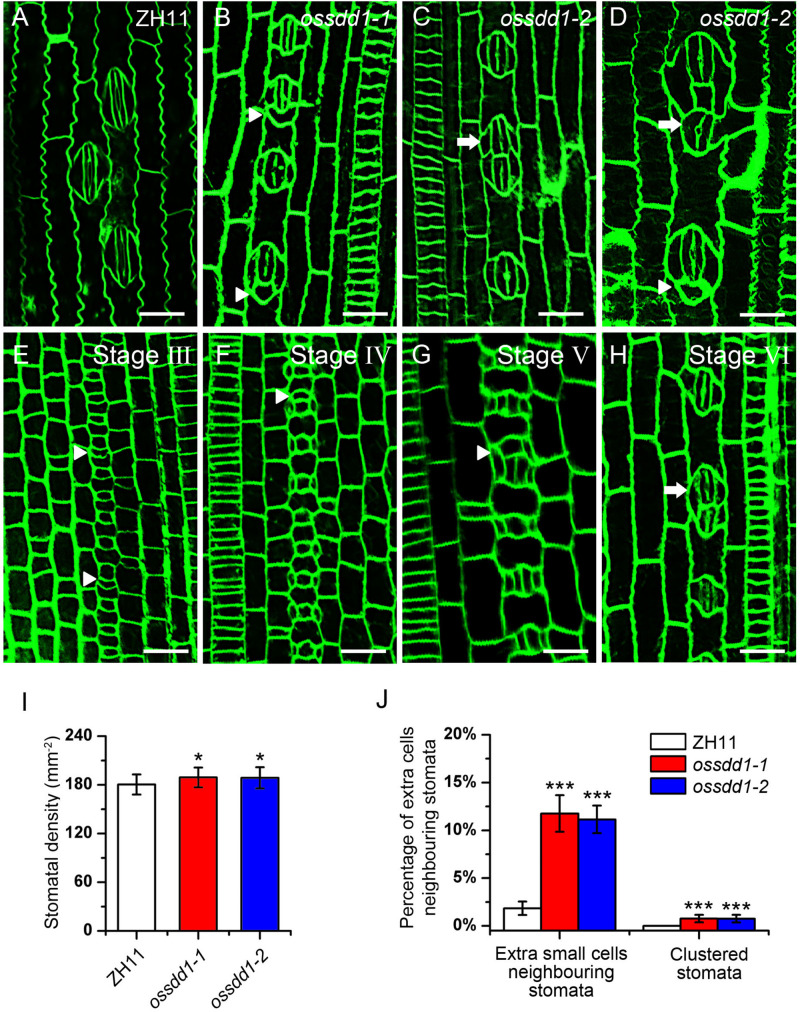
Phenotypic analysis of *ossdd1-1* and *ossdd1-2* mutants. **(A)** Mature stomata of ZH11. **(B)** An extra small cell neighboring stoma in *ossdd1-1*. **(C)** Two-cluster stomata with four subsidiary cells in *ossdd1-2*. **(D)** Five-cell stomatal complex and ectopic stomatal patterning in *ossdd1-2*. **(E)** The extra division of large sister cell resulting in an extra small cell neighboring GMC at stage III in *ossdd1-1*. **(F)** GMC with a small cell at stage IV in *ossdd1-1*. **(G)** Stomata with a small cell sharing SC at stage V. **(H)** Morphogenesis and differentiation of clustered stomata at stage VI. Bars, 10 μm. The white arrowheads indicated extra small cells neighboring stomata. The white arrows indicated clustered stomata. **(I)** Quantification of the stomatal density at mature stage. **(J)** Quantification of the clustered stomata and extra small cells neighboring stomata at the fifth leave. The error bars indicated the mean ± SEM, *n* = 10; ***P < 0.001; *0.01 < *P* < 0.05 by Student’s *t*-test.

The significant downregulation of *OsSDD1* in *rsd1* mutants led us to investigate the role of *OsSDD1* in stomatal development. There was one copy of *SDD1* in rice named *OsSDD1* (Supplementary Figure S7). To explore the phenotype of *OsSDD1*, CRISPR/Cas9 genome editing was performed, and we obtained two mutants named *ossdd1-1* and *ossdd1-2* (Supplementary Figure S8). In *ossdd1-1*, 1 bp was inserted at nucleotide position between 131 and 132, resulting in a PTT. In *ossdd1-2*, the 131st nucleotide was deleted, which also led to a PTT (Supplementary Figure S8). We then observed the stomatal phenotype in mature leaves of *ZH11*, *ossdd1-1*, and *ossdd1-2* (Figures 6A–D). Similar to *rsd1* mutants, both *ossdd1-1* and *ossdd1-2* exhibited clustered stomata and extra small cell neighboring stomata (Figures 6B–D,J). In addition, we observed the stomatal development process in *ossdd1-1* (Figures 6E–H). Similar to *rsd1*, an extra asymmetric division was observed in some larger sister cells of the *ossdd1* mutants at stage III, producing an extra small cell neighboring GMC (Figure 6E). A few of extra small cells neighboring GMC could induce the formation of SC at Stage V (Figure 6G) and may finally divide equally to form paired GCs, resulting in clustered stomata at stage VI (Figure 6H). The stomatal density of *ossdd1* mutants was slightly increased (Figure 6I), but the stomatal files had nothing different with ZH11 (Supplementary Figure S9). These observations suggested that *OsSDD1* is required for restricting ectopic ACDs and clustered stomata but not needed for stomatal file density in rice.

### *rsd1* Mutants Enhance the Dehydration Avoidance

**FIGURE 7:**
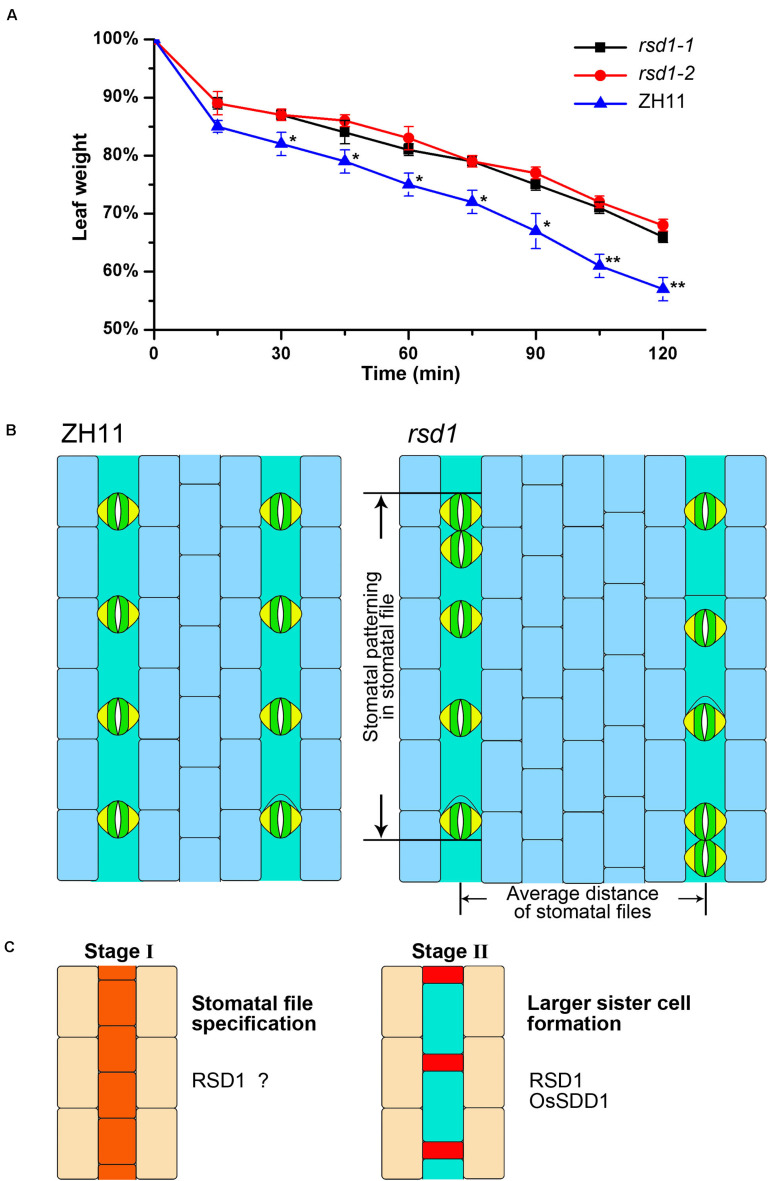
The *rsd1* mutants increase dehydration avoidance, and the model showed *RSD1* and *OsSDD1* regulating stomatal development. **(A)** Water loss in 8 weeks old detached leaves at different time points with three replicates. Three independent experiments were performed with similar results. The error bars indicated the mean ± SEM, *n* = 3; **0.001 < *P* < 0.01, *0.01 < *P* < 0.05 by Student’s *t*-test. **(B)** The stomatal patterning in ZH11 and *rsd1* mutants. **(C)** RSD1 and OsSDD1 required at early stage of stomatal development.

As stomatal density was decreased in *rsd1* mutants (Figure 1M), we performed a dehydration shock stress assay to determine the role of *RSD1* in water deficit. In ZH11, the fresh weight was reduced to 56.62% after 2 h of water deficit shock. Compared with ZH11, the water loss rate of *rsd1* mutants was significantly lower. The fresh weight of *rsd1-1* and *rsd1-2* was reduced to 65.57 and 67.94%, respectively, under the same treatment (Figure 7A). This result suggested that *RSD1* is required for regulating water loss by modulating stomatal density.

## Discussion

Stomata are key determinants of the trade-off between photosynthetic carbon fixation and water transpiration (Franks and Farquhar, 1999). Grasses with lower stomatal density have higher water use efficiency and greater drought tolerance than other species (Hughes et al., 2017). However, the detailed mechanisms of stomatal development in grasses were still very poorly understood. By forward genetic approach, we found *RSD1*, a new stomatal patterning regulatory gene, was required for inhibiting clustered stomata and promoting stomatal density (Figure 7B). Knockout of *OsSDD1* also produced clustered stomata and extra small cells adjacent to the stomata. *OsSDD1* and *RSD1* are both required for inhibiting ectopic ACDs and clustered stomata (Figure 7C).

In grasses, stomatal patterning is established when each stomatal precursor in a cell file divides once asymmetrically in the same orientation to produce a GMC (Facette and Smith, 2012). Therefore, there may be two ways to produce clustered stomata. One is the disruption of the division direction in neighboring stomatal precursors; another is the reentry of stomatal development in large sister cells to produce a stoma neighboring preexisting stoma. The *rsd1* mutants produce clustered stomata. Closer observation of stomatal development stages in the mutants showed that the direction of entry division was not affected. We found that the larger sister cell underwent an asymmetric division neighboring preexisting stoma or GMC, suggesting that the *RSD1* is required for inhibiting reentry division in larger sister cells. Similar causes of clustered stomatal were also observed in *ossdd1* mutants, suggesting that *OsSDD1* is also involved in preventing larger sister cells from dividing asymmetrically and promoting the differentiation of larger sister cells into pavement cells. In addition, some GMCs arrested and failed to differentiate into GC in *rsd1* mutants, suggesting a role of *RSD1* in promoting GMC to differentiate into mature stomata. These results suggest that *RSD1* promoted cell differentiation of both large sister cell and GMC.

The molecular mechanism of clustered stomata in grasses is still unknown. In *Arabidopsis*, *AtSPCH* and *AtICE1/AtSCRM2* coordinately established stomatal fate (MacAlister et al., 2007; Kanaoka et al., 2008). When they accumulated, additional stomata will be produced, and all epidermal cells will be turned into stomata in extreme conditions (MacAlister et al., 2007; Kanaoka et al., 2008). The upstream MAPK and receptor ligand signals ensure that additional stomata are not produced by limiting the accumulation of fate determination transcription factors (Bergmann et al., 2004; Lampard et al., 2008; Putarjunan et al., 2019). In grass plants, it is found that overexpression of stomatal fate factor *SPCH1/2* in *B. distachyon* could induce additional cell division in epidermal cells (Raissig et al., 2016; Wu et al., 2019). In POSTECH insertion mutant line of *osspch2*, the clustered stomata had been observed (Liu et al., 2009). Overexpression of *ICE1* and *SCRM2* produced only a small amount of extra cell division, whereas *Ubipro:BdICE1^*scrmD*^* produced clustered stomata (Raissig et al., 2016). In addition, the absence of *BdYODA1* leads to the disorder of cell fate in stomatal files, which results in clustered stomata (Abrash et al., 2018). In the *rsd1-1* mutant, the expression of *ICE1* and *EPFL9* was slightly up-regulated, which might be one of the reasons of clustered stomata.

*AtSDD1* is expressed in pseudo-meristem cells and GMCs, and the stomatal density of the mutant with functional deletion increased by 2–4 times and clustered, and its overexpression inhibited stomatal differentiation and decreased stomatal density (Berger and Altmann, 2000; Von Groll et al., 2002). Its function is to regulate stomatal development in the upstream of TMM but independent of EPFL gene family (Von Groll et al., 2002). Its protein function is conserved to a certain extent and has a significant effect on stomatal density in tomato and maize (Liu et al., 2015; Morales-Navarro et al., 2018). However, the function of *OsSDD1* in stomatal development is still unclear in rice. By constructing the knockout lines, we found that the *ossdd1* mutants have clustered stomata and extra small cells neighboring stomata (Figures 6B–D). The phenotype of *ossdd1* mutants is similar to *rsd1-1*. *OsSDD1* and *RSD1* are both required for inhibiting ectopic ACDs and clustered stomata.

In grasses, stomata are always arranged parallel and adjacent to leaf veins (Zwieniecki and Boyce, 2014; Nunes et al., 2020). The density of stomatal files was different in different development stages, species, or growth conditions (Stebbins and Shah, 1960). Therefore, the density of stomatal files is the key factor for stomatal density. The grass SHR/SCR is a common module that not only controls vein development and Kranz anatomy in maize (Slewinski et al., 2014; Hughes et al., 2019) but also regulates stomatal development in rice (Kamiya et al., 2003; Schuler et al., 2018; Wu et al., 2019). The deletion of *OsSHRs* will lead to the decrease of stomatal density in rice (Wu et al., 2019), while the overexpression of *ZmSHRs* in rice produces additional stomatal files far away from the vein to increase stomatal density (Schuler et al., 2018). In the *rsd1-1* mutant, we observed a decrease in the density of stomatal files in the leaf at the seedling stage (Figure 1L). However, the expression of *OsSHR* and *OsSCR* was not significantly changed in the *rsd1* mutants compared with ZH11 (Figure 5), suggesting that the decrease of stomatal files in the mutants is independent of *OsSHR*/*OsSCR*.

Rice is one of the most important food crops in the world. Although global climatic variability is a serious threat to food security, genetic engineering of stomatal development will enable us to create stress-tolerant crops (Serna and Fenoll, 2002; Korres et al., 2017). By controlling stomatal development and reducing stomatal density, rice can control water loss and make it easier to survive under drought conditions (Buckley et al., 2019). The lack of *RSD1* led to a reduction of stomatal density and the leaf water loss rate in rice. The effect of stomatal density on plants has been applied to create drought-resistant crops (Buckley et al., 2019). Recent research has shown that excessive expression of *EPF* genes in wheat and rice can significantly improve water use efficiency without affecting plant yield when stomatal density is reduced (Caine et al., 2019; Dunn et al., 2019). Therefore, RSD1 can be used as a candidate gene for breeding of drought-resistant rice.

## Data Availability Statement

The original contributions presented in the study are included in the article/Supplementary Material, further inquiries can be directed to the corresponding author.

## Author Contributions

QY and SH designed the experiments, and wrote the manuscript. QY, LC, WZ, TL, YA, ZW, YW, YX, and LY performed experiments, QY, LC, and SH revised the manuscript. All authors contributed to the article and approved the submitted version.

## Conflict of Interest

The authors declare that the research was conducted in the absence of any commercial or financial relationships that could be construed as a potential conflict of interest.

## Footnotes

1 http://www.gramene.org/

2 https://itol.embl.de/

3 http://skl.scau.edu.cn/
