# Supplementary material for: RSD1 Is Essential for Stomatal Patterning and Files in Rice
Source: Front Plant Sci. 2020 Nov 30;11:600021. doi: 10.3389/fpls.2020.600021 (PMC7733971; doi:10.3389/fpls.2020.600021)
Supplement: Supplementary file 1 [file Table_1.DOCX]

**Supplementary Information**


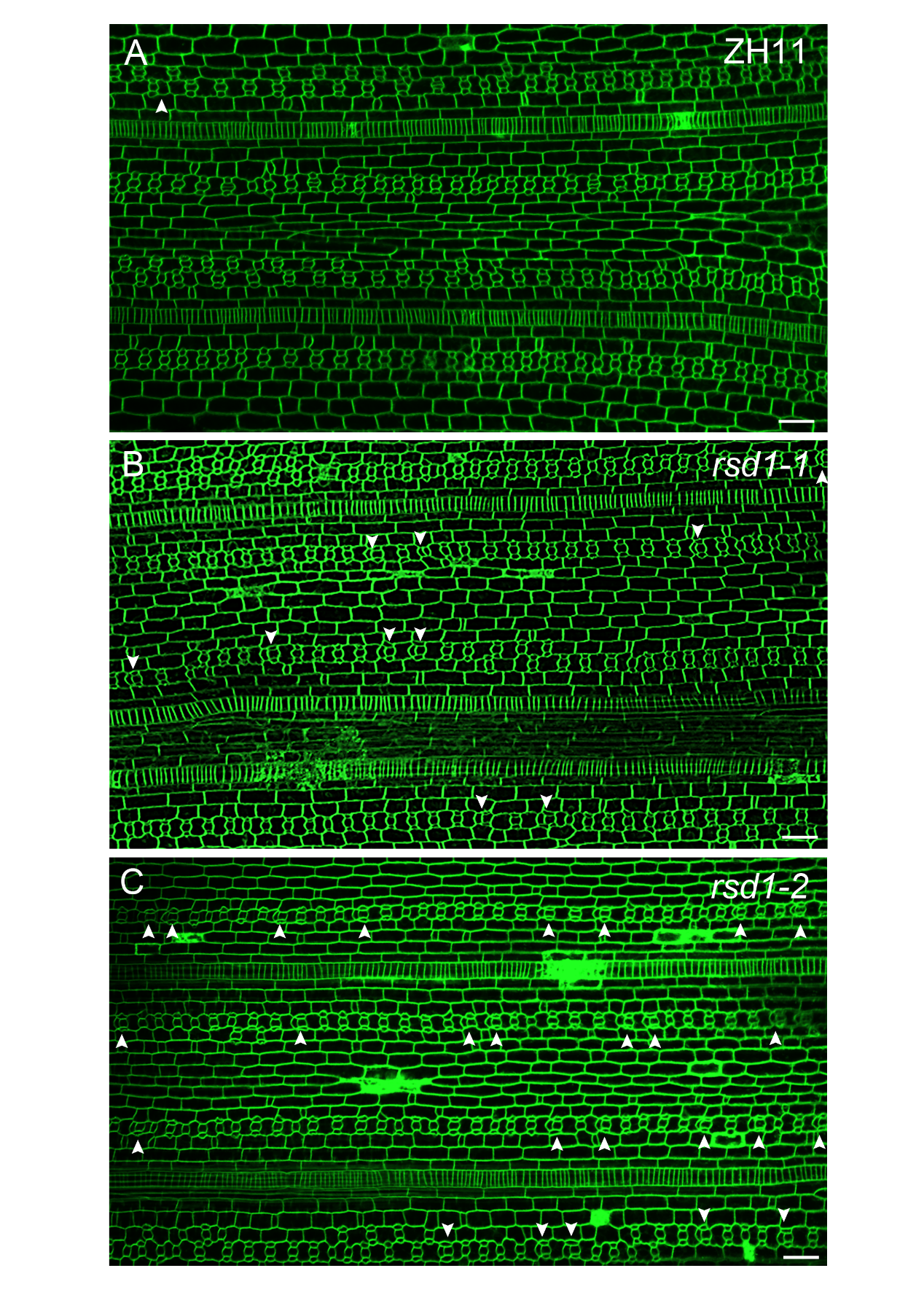


**Supplementary Figure 1. The extra small cells neighbouring GMCs in *rsd1* mutants at stage Ⅳ. (A)** Extra small cells of ZH11. **(B)** Extra small cells of *rsd1-1*. **(C)** Extra small cells of *rsd1*-2. Bars, 20 μm. The white arrowheads indicated ectopic extra small cells neighbouring GMCs.


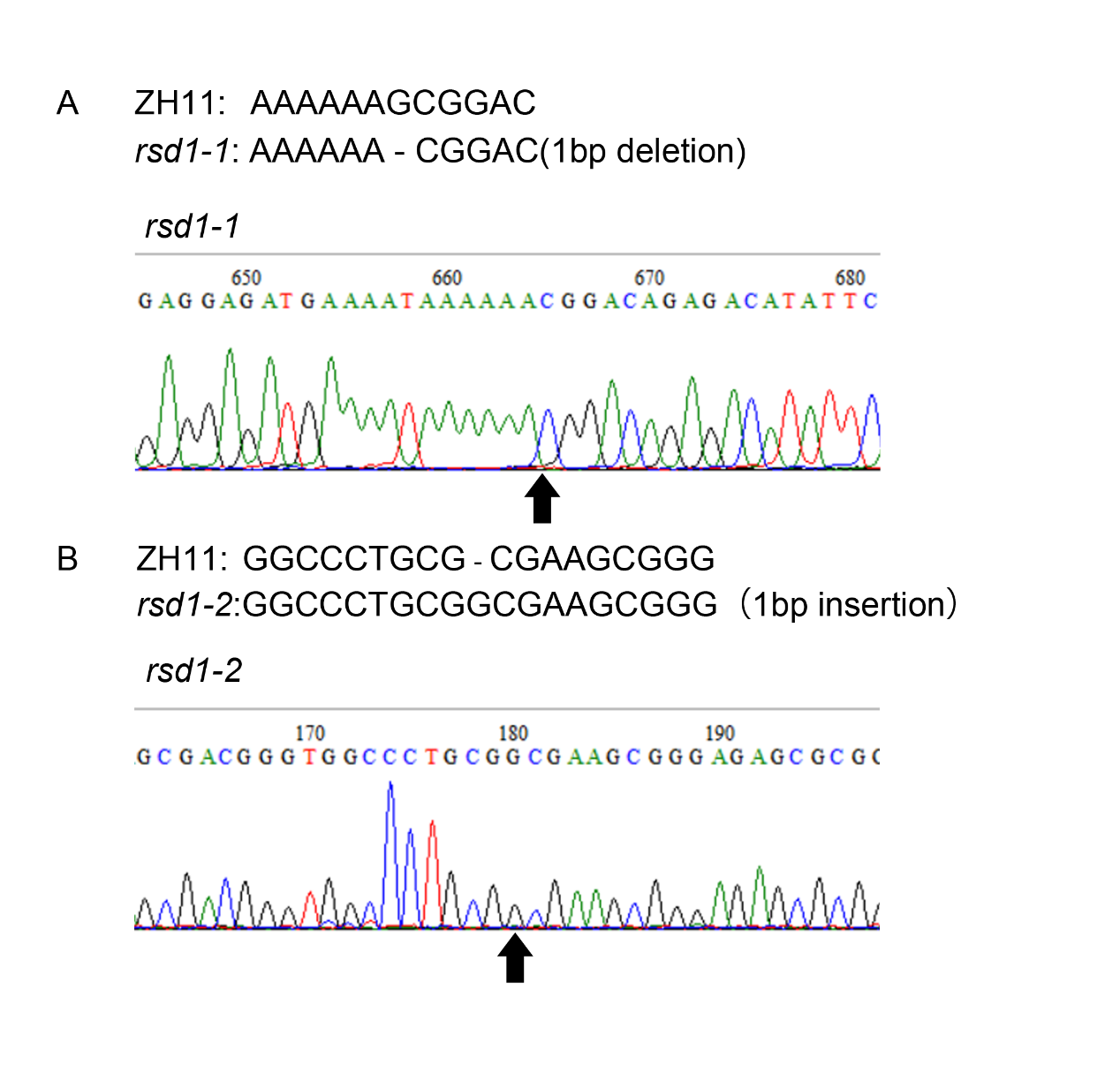


**Supplementary Figure 2. Genotyping of *rsd1* mutants. (A)** Chromatograms of *rsd1-1*. **(B)** Chromatograms of CRISPR-induced mutations of *rsd1-2*. The arrows indicated the mutation sites.


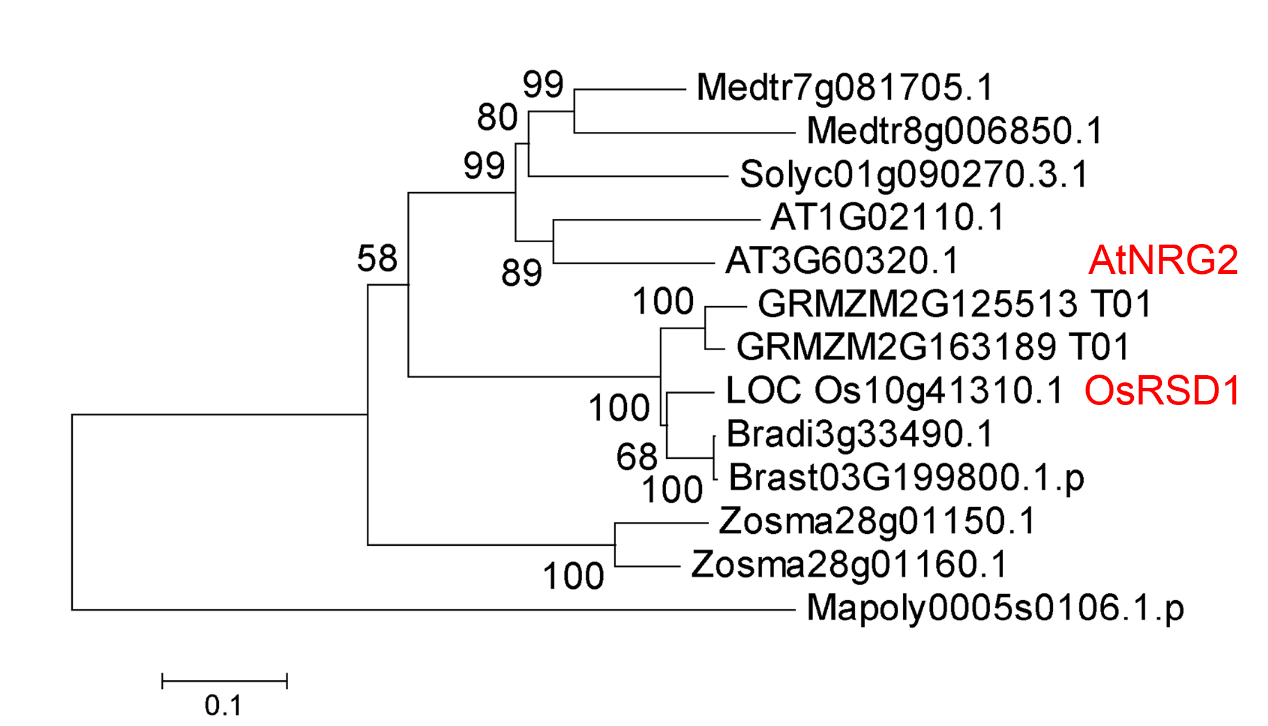


**Supplementary Figure 3. Phylogenetic tree of *RSD1* in 10 plants.** The phylogenetic tree was constructed using amino acid sequences of *Marchantia polymorpha*, *Arabidopsis thaliana*, *Medicago truncatula*, *Solanum lycopersicum*, *Oryza sativa*, *Zea mays*, *Brachypodium distachyon*, *Sorghum bicolor*, *Zostera marina* and *Brachypodium stacei* based on Phytozome V12.1, using the Neighbor-Joining method in MEGA 5. Bootstrap values for 1000 replicates were given in nodes as percentages.


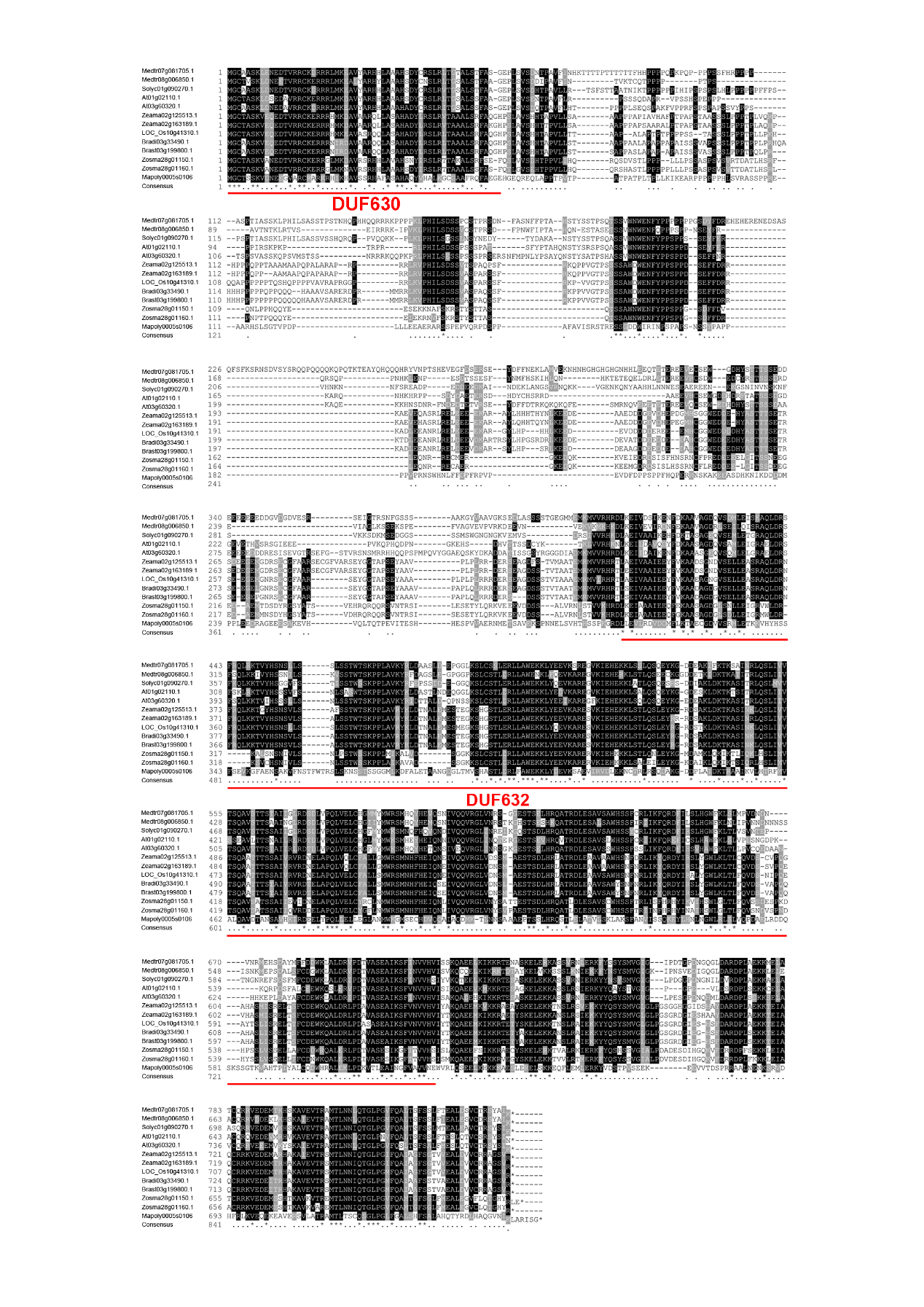


**Supplementary Figure 4. Multiple sequences alignment of *RSD1*.** A Multiple sequences alignment of 13 proteins. The amino acids showing identity were shaded black, whereas similar amino acids were shaded grey. The DUF630 and DUF632 domain were labeled in red line.


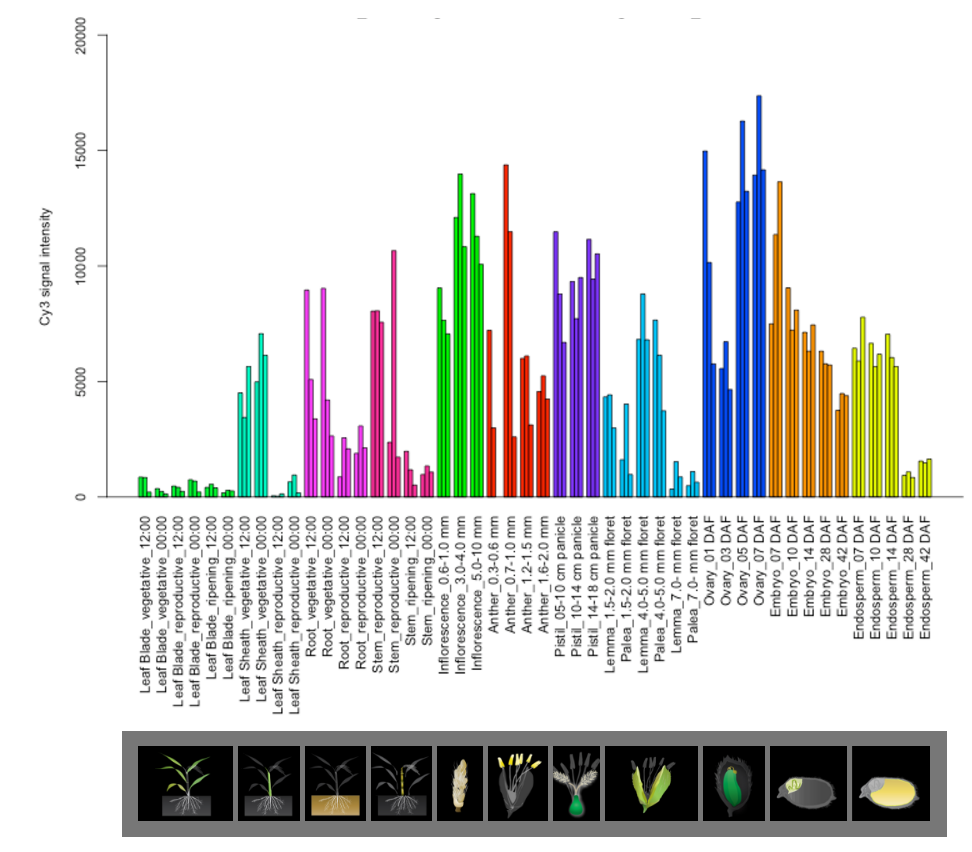


**Supplementary Figure 5. Expression analysis of *RSD1* in different tissue.** The expression file was acquired from RiceXPro (https://ricexpro.dna.affrc.go.jp/category-select.php).


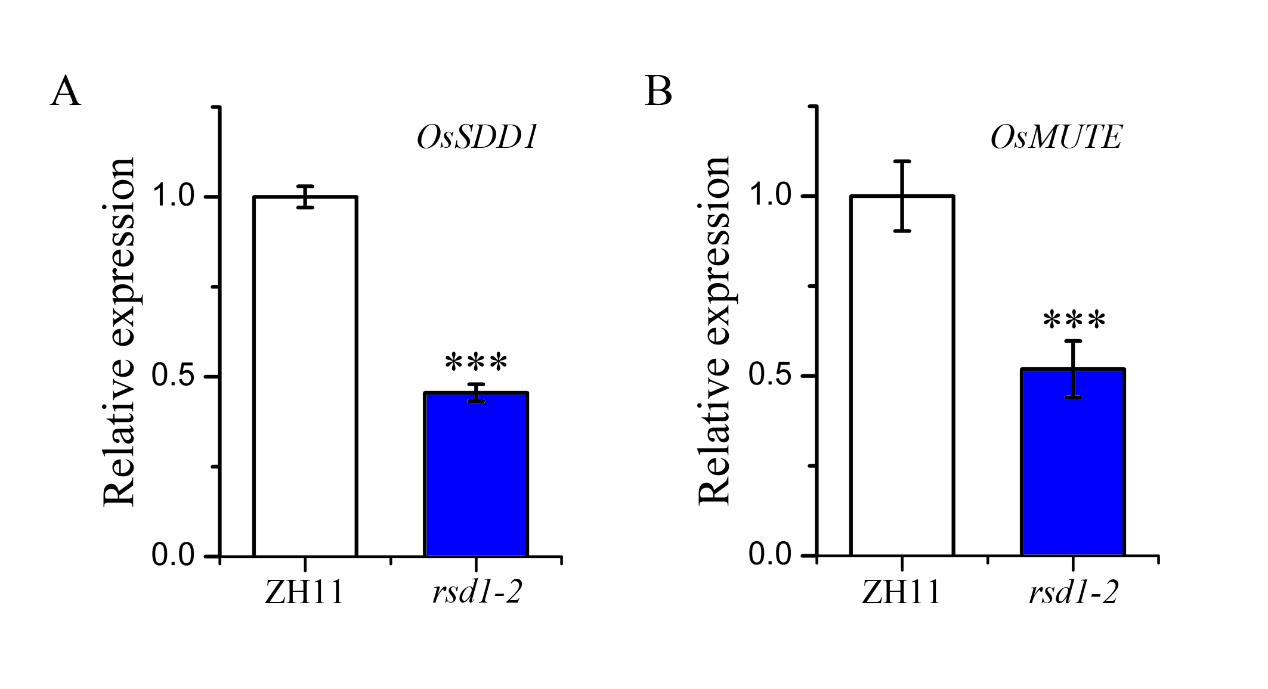


**Supplementary Figure 6. Expression analysis of *OsMUTE* and *OsSDD1* in *rsd1-2* mutant. (A)** Analyzing the expression of *OsSDD1* by RT-qPCR. **(B)** Analyzing the expression of *OsMUTE* by RT-qPCR. OsUBQ10 was used as the internal control. The error bars indicated mean ± SD, n = 3; ***, P < 0.001. **, 0.001 < P < 0.01. *, 0.01 < P < 0.05 by Student’s t-test.


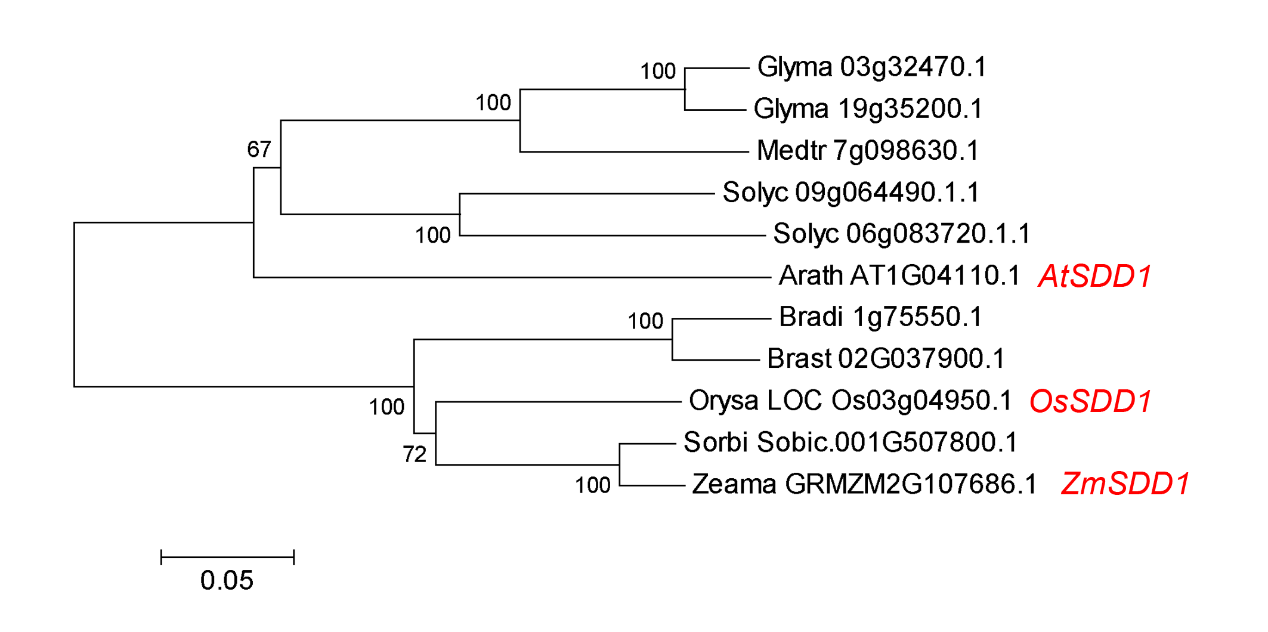


**Supplementary Figure 7. Phylogenetic tree of *SDD1* in 9 land plants.** The phylogenetic tree was constructed using amino acid sequences of *Arabidopsis thaliana*, *Glycine max*, *Medicago truncatula*, *Solanum lycopersicum*, *Oryza sativa*, *Zea mays*, *Brachypodium distachyon*, *Sorghum bicolor* and *Brachypodium stacei* based on Phytozome V12.1, using the Neighbor-Joining method in MEGA 5. Bootstrap values for 1000 replicates were given in nodes as percentages.


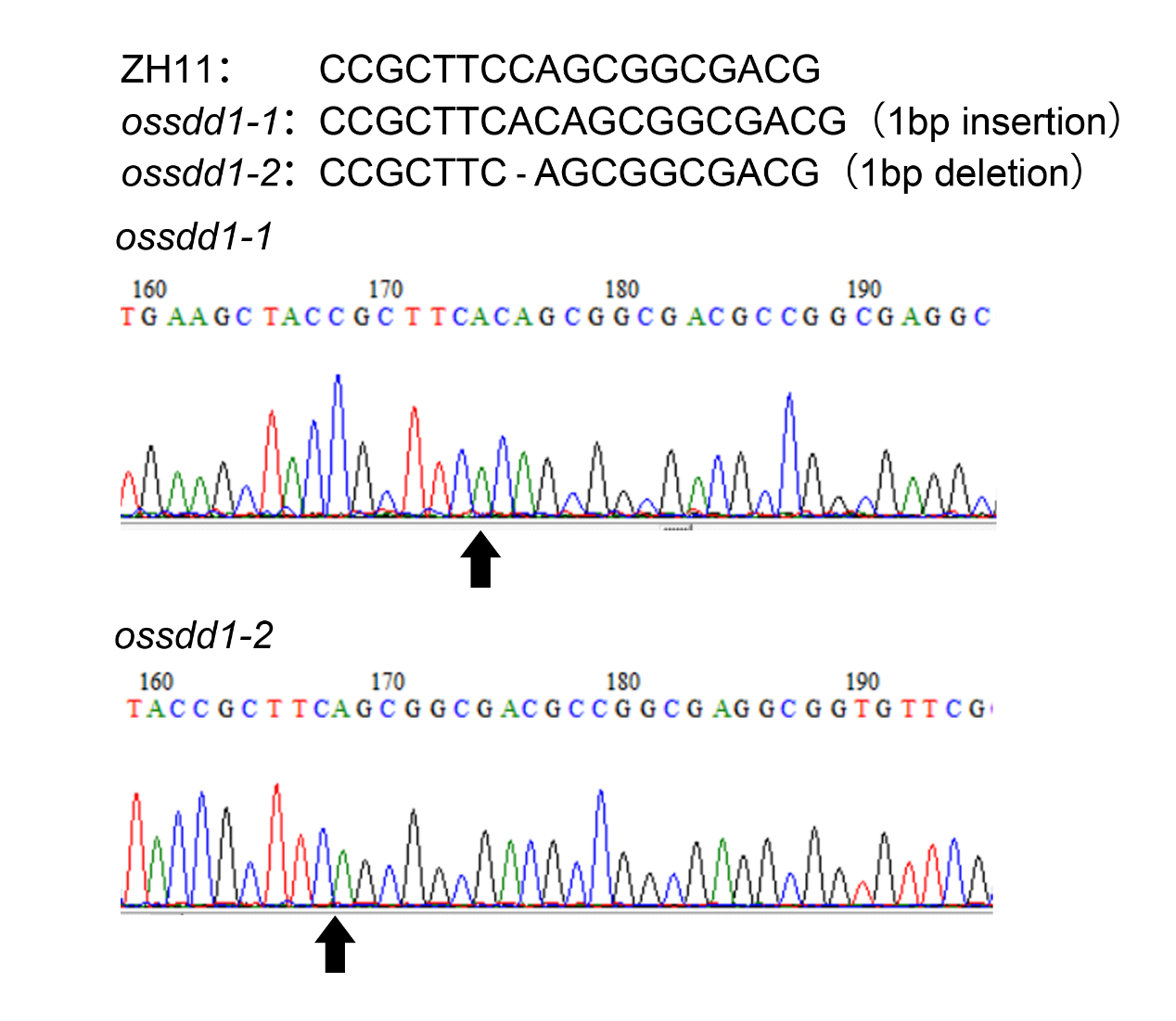
 **Supplementary Figure 8. Genotyping of *ossdd1* mutants.** Chromatograms of CRISPR-induced mutations of *ossdd1* mutants. The arrows indicated the mutation sites.


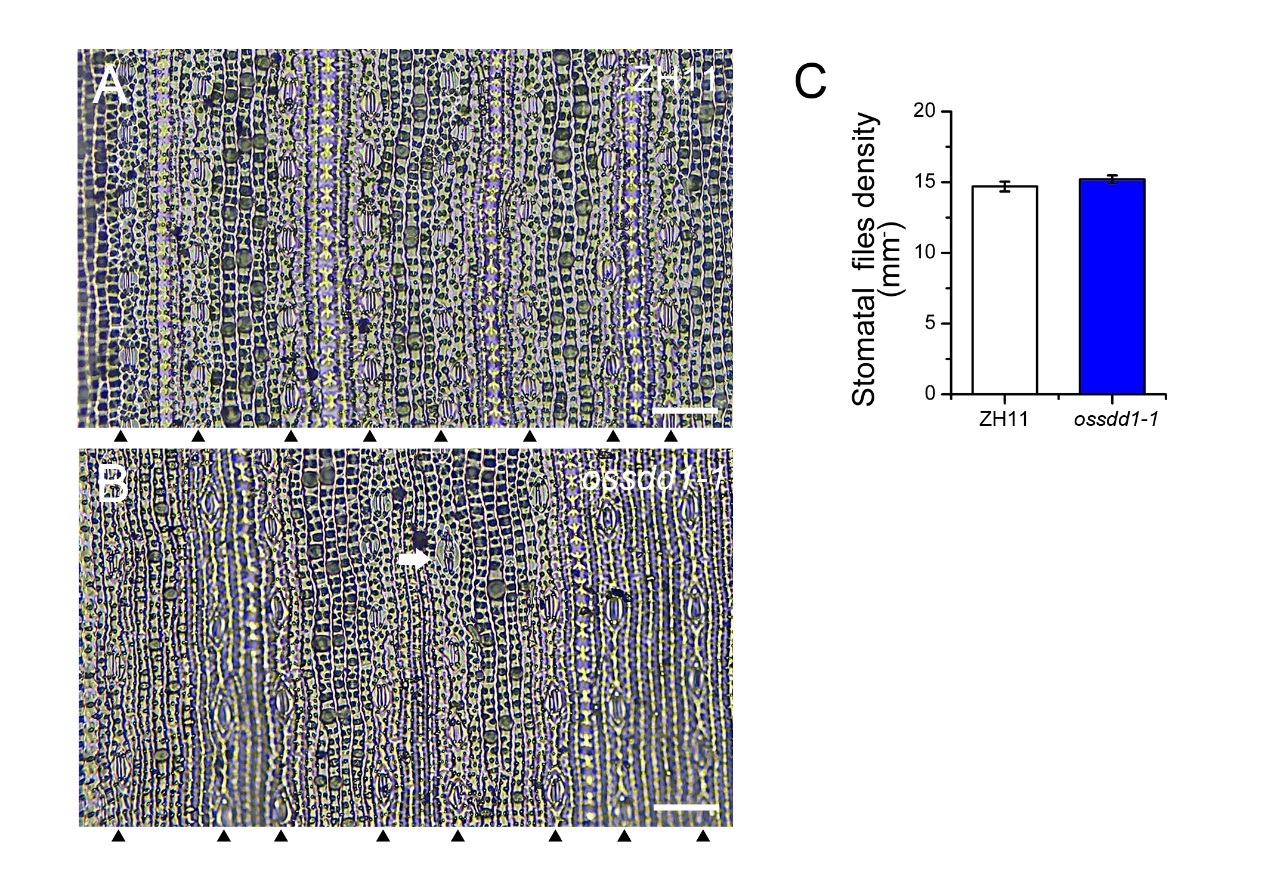


**Supplementary Figure 9. The *ossdd1-1* mutant showed normal stomatal files. (A)** Mature stomata files of ZH11. **(B)** Mature stomata files of *ossdd1-1*. Bars, 50 μm. The white arrows indicated clustered stomata. **(C)** Quantification of the density of stomatal files at the fifth leaf. The error bars indicated the mean ± SEM, N = 20; ***, *P* < 0.001 by Student’s *t*-test.

**Supplementary Table 1.** Primer sequences

| Primer | Forward primer | Reverse primer | Use |
| --- | --- | --- | --- |
| 10-01 | TTATGTCAACCTTACAATT | GCATACTGTATTCCTATATC | Map-based cloning |
| 10-02 | TTGCTTAGTGGTAAGCTATG | GGAGTTCTTGAAGAAACTCC |  |
| 10-03 | GTATGTTTGTGGAGTAATAT | CATGCAATGTGCCAAGAACC |  |
| 10-04 | GCATACGAATGTACTTGTGT | TGGAAGTATGCGTGGAATAT |  |
| 10-05 | CTCAAATCACTTATATTATG | GTGTAACCAGTAGAAGAATG |  |
| 10-06 | TGTATAATTTGTTGAGATGAG | GTAGATCTTGTGATATTGGAG |  |
| 10-07 | CACACGTGCAACTAGACATG | ACAATGCTATCTGTATCTGAC |  |
| X-01 | GATTCACGGGTCGATCTACT | GTGTGGTGCCATGCCTAATG |  |
| X-02 | CAAGTATAATAATACTTAGAG | GAGCAAGGCTAATAATACATC |  |
| X-08 | GTTGAAGTGCTACAGGTATG | GCTTCCTCAGCCTTGATCTC |  |
| X-09 | GTATTCGCACTCTCAAATGAG | CAAATTAAGTCACAGATAAGTG |  |
| X-10 | CAAGAATTCTAAGAGCTCG | CGAGCTTATGCATAAGCTTTG |  |
| X-11 | CTACATAAAACCTCGAATCC | CACCTGTCGGCGTCAGTCAC |  |
| X-12 | TAGGAGGAGCAGAAGGAATAG | CGATCGATCGTCTTCTTCCAC |  |
| *rsd1-1* | AGCCTGGAGTACAGAGGGAGG | TAACGAACGGTTTCTTGGAAAG | Mutant identification |
| *rsd1-2* | TGGGGGGCGATGGGGTGC | GAGGCGGCTGATGCGACTGCG | CRISPR/Cas9 identification |
| *rel2* | CCTCAAACTGTGTAGTCACAACCTT | GCTGGAGTCGGACAGGATGT | Mutant identification |
| *ossdd1-1*/*2* | GCAATTTGCAAGCTAGAGCGA | CAGAAGTCGAGCCCCAAGAA | CRISPR/Cas9 identification |
| qRT-OsSPCH1 | CAATAACAAGCGGCGGGAGG | TGAGCACCTGGTCGGTGTAG | qRT-PCR primer for OsSPCH1 |
| qRT-OsSPCH2 | TCGAGATCCTGCACGTCAGCATGC | GCACCAGCTCCTCTGCACTCAGC | qRT-PCR primer for OsSPCH2 |
| qRT-OsMUTE | TAATCTCGCCGGCATCGATATC | CTGCTGCTTATTTTCGTCCTTG | qRT-PCR primer for OsMUTE |
| qRT-OsFAMA | CTACTTCCTCAGGTTCCAGTC | GCTCGTCAACGACCTGAT | qRT-PCR primer for OsFAMA |
| qRT-OsICE1 | TCAGTTGCTTCAATGGCTTTAC | AACTTCCTTCTGTCGAAAATGC | qRT-PCR primer for OsICE1 |
| qRT-OsICE2 | ATGAACTTGAGTCTGCTCCTAG | TGGATATTGACTGCATGACCTT | qRT-PCR primer for OsICE2 |
| qRT-OsFLP | TAGCAGCATCGGAGAATCTTAG | GTGTTTCTGCATTTGCTTCAAC | qRT-PCR primer for OsFLP |
| qRT-OsYODA1 | CTGCAATGTTCAAGATCGGAAA | TAACAGCCAAATGTTCCAATGG | qRT-PCR primer for OsYODA1 |
| qRT-OsYODA2 | AAAAGGGGAGAACAAAATCACG | CAAAACGACCTTTGATTCGGTA | qRT-PCR primer for OsYODA2 |
| qRT-OsSDD1 | CTCTTGTTCTTCTGCCTGTTTC | GGACTTCTCAAGAAACGAGAGA | qRT-PCR primer for OsSDD1 |
| qRT-OsEPF1 | CCCACTGGAGTACGTACTAATC | ATAATGAACGAAATGTGGGCAC | qRT-PCR primer for OsEPF1 |
| qRT-OsEPF2 | CATCCTTCTCATACTCGCAGTC | TTGAAGCTCACCATGACGC | qRT-PCR primer for OsEPF2 |
| qRT-OsEPFL9 | AATATGCACATACAACGAGTGC | TACTAGCTGGCCACTTGTTAAA | qRT-PCR primer for OsEPFL9 |
| qRT-OsPAN2 | GTGTTCGTCAATCTGACTATGC | CTCCGGAGATTGCCTATGTTAT | qRT-PCR primer for OsPAN2 |
| qRT-OsSCR1 | TCA TCCGACCTTGTAACCCCG | AGCAGCGTCAGCAGGTGGAG | qRT-PCR primer for OsSCR1 |
| qRT-OsSCR2 | GCGAGTTACAGCGAGGATAGC | CAAGCAGAGGCAGTGAGAAGG | qRT-PCR primer for OsSCR2 |
| qRT-OsSHR1 | GTACCAAGAAGAATGCGGCAAC | AGCTCGTTGAGCATCCACATG | qRT-PCR primer for OsSHR1 |
| qRT-OsSHR2 | ACCCTCTTCAGGTTGGTTAGCC | GTGGAACTGGCGGGAGGAAG | qRT-PCR primer for OsSHR2 |
| qRT-OsUBQ10 | TGGTCAGTAATCAGCCAGTTTGG | GCACCACAAATACTTGACGAACAG | qRT-PCR primer for OsUBQ10 |

**Supplementary Table 2.** The targeting sequences of CRISPR/Cas9 lines

| Mutant name | sgRNA+PAM |
| --- | --- |
| *rsd1-2* | GCGCTCTCCCGCTTCGCGCAGGG |
| *ossdd1-1*/*2* | CATGAAGCTACCGCTTCCAGCGG |
